# Whole-genome Sequencing Provides Data for Stratifying Infection Prevention and Control Management of Nosocomial Influenza A

**DOI:** 10.1093/cid/ciz020

**Published:** 2019-04-17

**Authors:** Sunando Roy, John Hartley, Helen Dunn, Rachel Williams, Charlotte A Williams, Judith Breuer

**Affiliations:** 1 Division of Infection and Immunity, University College London, United Kingdom; 2 Great Ormond Street Hospital for Children, United Kingdom; 3 Infection, Immunity, Inflammation and Physiological Medicine, Institute of Child Health, University College London, United Kingdom

**Keywords:** next-generation sequencing, influenza, infection control, transmission, whole genome

## Abstract

**Background:**

Influenza A virus causes annual epidemics in humans and is associated with significant morbidity and mortality. Haemagglutinin (HA) and neuraminidase (NA) gene sequencing have traditionally been used to identify the virus genotype, although their utility in detecting outbreak clusters is still unclear. The objective of this study was to determine the utility, if any, of whole-genome sequencing over HA/NA sequencing for infection prevention and control (IPC) in hospitals.

**Methods:**

We obtained all clinical samples from influenza (H1N1)-positive patients at the Great Ormond Street Hospital between January and March 2016. Samples were sequenced using targeted enrichment on an Illumina MiSeq sequencer. Maximum likelihood trees were computed for both whole genomes and concatenated HA/NA sequences. Epidemiological data was taken from routine IPC team activity during the period.

**Results:**

Complete genomes were obtained for 65/80 samples from 38 patients. Conventional IPC analysis recognized 1 outbreak, involving 3 children, and identified another potential cluster in the haemato-oncology ward. Whole-genome and HA/NA phylogeny both accurately identified the previously known outbreak cluster. However, HA/NA sequencing additionally identified unrelated strains as part of this outbreak cluster. A whole-genome analysis identified a further cluster of 2 infections that had been previously missed and refuted suspicions of transmission in the haemato-oncology wards.

**Conclusions:**

Whole-genome sequencing is better at identifying outbreak clusters in a hospital setting than HA/NA sequencing. Whole-genome sequencing could provide a faster and more reliable method for outbreak monitoring and supplement routine IPC team work to allow the prevention of transmission.

## Teaser

This study shows the usefulness of whole-genome sequencing over single-gene sequencing to monitor the transmission of influenza in a hospital setting. We were able to identify direct transmission clusters that were previously missed by infection prevention and control.

Influenza A causes seasonal epidemics, as well as sporadic, large-scale pandemics, in human hosts, with associated high levels of mortality and morbidity [1]. In immunocompromised and immunosuppressed children, the risk of mortality is even higher [2]. These patients also tend to shed the virus over a prolonged period, leading to higher chances of transmission [3, 4]. For this reason, it is important to monitor transmission in a hospital setting housing both immunocompromised and immunosuppressed patients.

Haemagglutinin (HA) and neuraminidase (NA) gene sequencing have been routinely used in hospitals to confirm influenza outbreak clusters among patients [5–7]. Gene sequencing also provides information on genotypes, subtypes, and drug resistance in a hospital setting [3]. In recent years, whole-genome sequencing (WGS) has been widely used to conduct outbreak investigations of a wide range of infectious pathogens [8–11], with a few studies focusing on the epidemiology of the influenza virus [12, 13]. A recent study concluded that, in the hospital setting, WGS did not provide any additional information on influenza outbreaks and transmission chains, compared to HA/NA gene sequencing alone [14]. This conclusion is attractive, as sequencing of the influenza HA and NA genes is routinely used for genotyping strains [3, 4]. Here, we tested the value of WGS as a clinical tool for the identification of potential nosocomial transmission, and we discuss whether it could be useful in real time.

## METHODS

Residual nasopharyngeal aspirate samples were collected from all patients who tested positive for influenza A (H1N1) at Great Ormond Street Hospital (GOSH) during January to March 2016. The study was approved under the University College of London Pathogen BioBank National Research Ethics Committee London–Fulham (Research Ethics Committee number 17/LO/1530)

Overall, 80 samples were sequenced at multiple time points from 38 individuals. Samples were sequenced using the Agilent SureSelect^XT^ (SSXT) targeted enrichment method, as previously described [15, 16]. Custom 120-mer RNA baits were designed using a comprehensive set of influenza A sequences. Bait sets were validated using clinical samples from multiple hospitals in the United Kingdom [17, 18]. Sequencing libraries were prepared using the 200ng input SureSelect XT protocol and sequenced on an Illumina MiSeq sequencer. Sequences were assembled using a reference-based pipeline in CLC Genomics Workbench version 8.03, and consensus sequences were extracted with a minimum of 10X coverage. Alignments were made using MAFFT 7.212 [19]. Maximum likelihood trees were computed for the sequenced strains, along with other database H1N1 strains, in PhyML 3.1 [20] with 500 bootstrap replicates and were visualized using FigTree 1.4.2 (http://tree.bio.ed.ac.uk/software/figtree/). Pairwise single-nucleotide variations were computed using the ape package (dist.dna) in R. Clinical data were obtained from a review of the patient records. The transmission network was constructed using PopART [21].

## RESULTS

The patient population comprised 38 children, 32 from GOSH and 6 as inpatients in other hospitals, with influenza A detected by polymerase chain reaction of nasopharyngeal aspirates (Table 1). Complete sequence data were obtained from 36 patients, while 2 patients failed sequencing. Routine epidemiology was obtained from infection prevention and control (IPC) team activity: 9 of the patients developed symptoms more than 48 hours after admission and were thus deemed to have health-care–associated infections. There were 3 children who were linked by IPC data on 1 ward (cases 25, 26, and 27; Table 1) and considered to be an outbreak. There were 2 children who had recently been on another ward (cases 27 and 35) and were considered as possibly linked by IPC methods. In addition, there were 11 patients (Patients 37, 39, 42, 43, 58, 59, 63, 70, 75, 76, and 77; Table 1) linked to the haematology/oncology/immunology/bone marrow transplant services, who shared some patient services and were suspected to have cross-infections, but a connection between those patients had not been conclusively demonstrated by conventional IPC.

**Table 1.** Clinical Details for Patients Sequenced as Part of the Study

Patient No	Ward	Service	Date of Admission or OP Visit	Specimen Collection Date	Location When Tested	Status When Sampled	Days IP Before Onset	Prior GOSH Contact if Not HCAI	Period Since Any Contact With the Hospital if Not HCAI	Linked and Possibly Linked Patient (Pairwise Distance) With All GOSH Strains	Comments
25^a^	R	Metabolic	26/08/2015	25/01/2016	IP	HCAI	152	NA	NA	26 (2), 27 (1)	Index case. Infected 26 and 27 on the same ward
26^a^	R	Endocrine	04/01/2016	26/01/2016	IP	HCAI	22	NA	NA	25 (2), 27 (1)	Same ward as 25 and 27
27^a^	R	Endocrine	04/01/2016	27/01/2016	IP	HCAI	23	NA	NA	25 (1), 26 (1)	Same ward as 25 and 26
53^b,c,d^	B	IPP	14/01/2016	15/01/2016	IP	POA	NA	Y	2 days	54 (1), 45 (7), 49 (8), 81 (8), 36 (9), 70 (10), 37 (11)	OPD 12/01/2016
54^b,c,e^	B	IPP	07/01/2016	15/01/2016	IP	HCAI	8	NA	NA	53 (1), 45 (6), 49 (7), 81 (7), 36 (8), 70 (9)	Mother unwell
36^b,d^	PI	ICU	31/01/2016	01/02/2016	IP	POA	NA	None	NA	37 (8), 54 (8), 53 (9), 70 (9), 45 (10), 49 (11)	New patient
37^b,d^	EL	Haem/onc	05/02/2016	05/02/2016	IP	POA	NA	Y	7 days	36 (8), 54 (10)	OPD 15/01/16
45^b^	EXT	External	None	11/02/2016	Ext	EXT	NA	Y	5 months	54 (6), 53 (7), 49 (9), 36 (10), 70 (11)	OPD 15/09/2015 referred sample
49^b,c,e^	PP	IPP/ENT	11/02/2016	22/02/2016	IP	HCAI	11	NA	NA	54 (7), 53 (8), 45 (9), 36 (11)	
70^b^	HAOPD	Haem/onc	11/03/2016	11/03/2016	OP	POPD	NA	None	NA	36 (9), 54 (9), 53 (10), 37 (11), 45 (11)	OPD and not admitted
81^b,e^	SQUIR	Surgical	15/09/2015	25/03/2016	IP	HCAI	192	NA	NA	54 (7), 53 (8)	
38^f^	PI	ICU	05/02/2016	05/02/2016	IP	POA	NA	Y	2 months	40 (7), 35 (23)	OPD 10/12/2015
40^f^	EXT	External	None	28/01/2016	Ext	EXT	NA	Y	1.5 months	38 (7), 35 (20)	OPD 18/12/2015 referred sample
43^g^	EL	Haem/onc	21/01/2016	09/02/2016	IP	HCAI	19	None	NA	36 (21), 54 (21)	New patient
58^g^	G	Haem/onc	24/01/2016	26/01/2016	IP	POA	NA	Y	3 days	36 (21), 54 (21)	OPD 23/01/16. OPD 21/01/2016.
39^g^	HAOPD	Haem/onc	29/01/2016	29/01/2016	OP	POA	NA	Y	2.5 months	35 (41), 36 (42), 54 (42)	HAOPD 6/11/2015
59^g^	EL	Haem/onc	25/02/2016	25/02/2016	IP	POA	NA	Y	3 days	35(90), 36 (90), 54 (90)	IP GOSH 16/2–22/2/16 parents were symptomatic.
63^g^	HAOPD	Haem/onc	02/03/2016	02/03/2016	OP	POA	NA	Y	20 days	35 (157), 42 (158)	HAOPD 12/02/2016
75^g^	HAOPD	Haem/onc	16/03/2016	16/03/2016	OP	POA	NA	Y	29 days	36(25), 54 (25)	HAOPD 15/02/2016
76^g^	HAOPD	Haem/onc	16/03/2016	16/03/2016	OP	POA	NA	Y	20 days	36 20), 54 (20)	HAOPD 24/02/2016,
77^g^	HAOPD	Haem/onc	16/03/2016	16/03/2016	OP	POA	NA	Y	19 days	36 25), 54 (25)	IP 23/02/2016 25/02/2016
35	EA	Renal	01/02/2016	01/02/2016	IP	POA	NA	Y	2 days	27 (13), 25 (14), 26 (14)	OPD 20 and 21/01/2016; admission 28/01-30/01/2016
62^f^	BU	IPP	01/03/2016	02/03/2016	IP	POA	NA	Y	27 days	53 (13), 54 (12)	IP 12–14/1/2016 OPD 2/02/2016
69	BA	Resp	06/03/2016	10/03/2016	IP	HCAI	4	Y	25 days	36 (12), 37 (14), 54 (14)	OPD 9/2/2016;
47^f^	PI	ICU	15/02/2016	16/02/2016	IP	POA	NA	Y	2 months	36 (12), 54 (12)	IP GOSH 22/11–8/12/2015;
60^f^	PI	ICU	28/02/2016	28/02/2016	IP	POA	NA	None	NA	36 (12), 54 (12)	New patient
79	P	Rheum	17/03/2016	18/03/2016	IP	POA	NA	Y	13 days	36 (13), 37 (15), 54 (15)	OPD 4/3/2016
42	EXT	External	None	01/02/2016	Ext	EXT	NA	Y	2 months	36 (15), 54 (15)	IP GOSH 21/10/2015; referred sample
73	EXT	External	None	12/03/2016	Ext	EXT	NA	None	NA	36 (17), 54 (17)	No GOSH contact; referred sample
74	K	Neurosurgical	14/03/2016	16/03/2016	IP	POA	NA	Y	2 months	36 (18), 54 (18)	OPD 19/01/2016,
51	PI	ICU	11/01/2016	12/01/2016	IP	POA	NA	Y	8.5 months	36 (22),53 (22), 54 (22)	OPD 27/04/2015
55^b^	SQ	Surgical	22/12/2015	18/01/2016	IP	HCAI	27	NA	NA	36 (23), 54 (23)	Father had a respiratory infection
34	EA	Renal	31/01/2016	31/01/2016	IP	POA	NA	Y	4 years	36 (23), 54 (23)	IP and OPD 2012.
61	EXT	External	None	01/03/2016	Ext	EXT	NA	None	NA	36 (22), 54 (22)	No GOSH contact; referred sample
78^f^	BU	IPP	18/03/2016	19/03/2016	IP	POA	NA	Y	10 days	36 (25), 54 (25)	OPD 21/12/2015–15/01/2016 OPD 3/3/16 and 8/3/3016
65	EXT	External	None	03/03/2016	Ext	EXT	NA	None	NA	51 (24), 36 (30), 54 (30)	No GOSH contact; referred sample

Abbreviations: ENT, ear nose throat; EXT, external sample referred for testing; GOSH, Great Ormond Street Hospital; Haem/Onc, hamatology/oncology; HAOPD, haematology/oncology outpatient department; HCAI, healthcare–acquired infection; ICU, intensive care unit; IP, inpatient; IPP, private patient; NA, not applicable; OP, outpatient; OPD, outpatient department; POA, positive on arrival; POPD, positive in outpatient department; Resp, respiratory; Rheum, rheumatology inpatient.

^a^Cluster 1 known outbreak.

^b^Cluster 2 and cases putatively linked to Cluster 2.

^c^Private patient, potentially sharing facilities and living accommodation.

^d^Attended OPD within 4 days of each other.

^e^Attended by the surgical team.

^f^Other putatively linked cases.

^g^Unlinked haemato-oncology cases.

**Figure 1.**
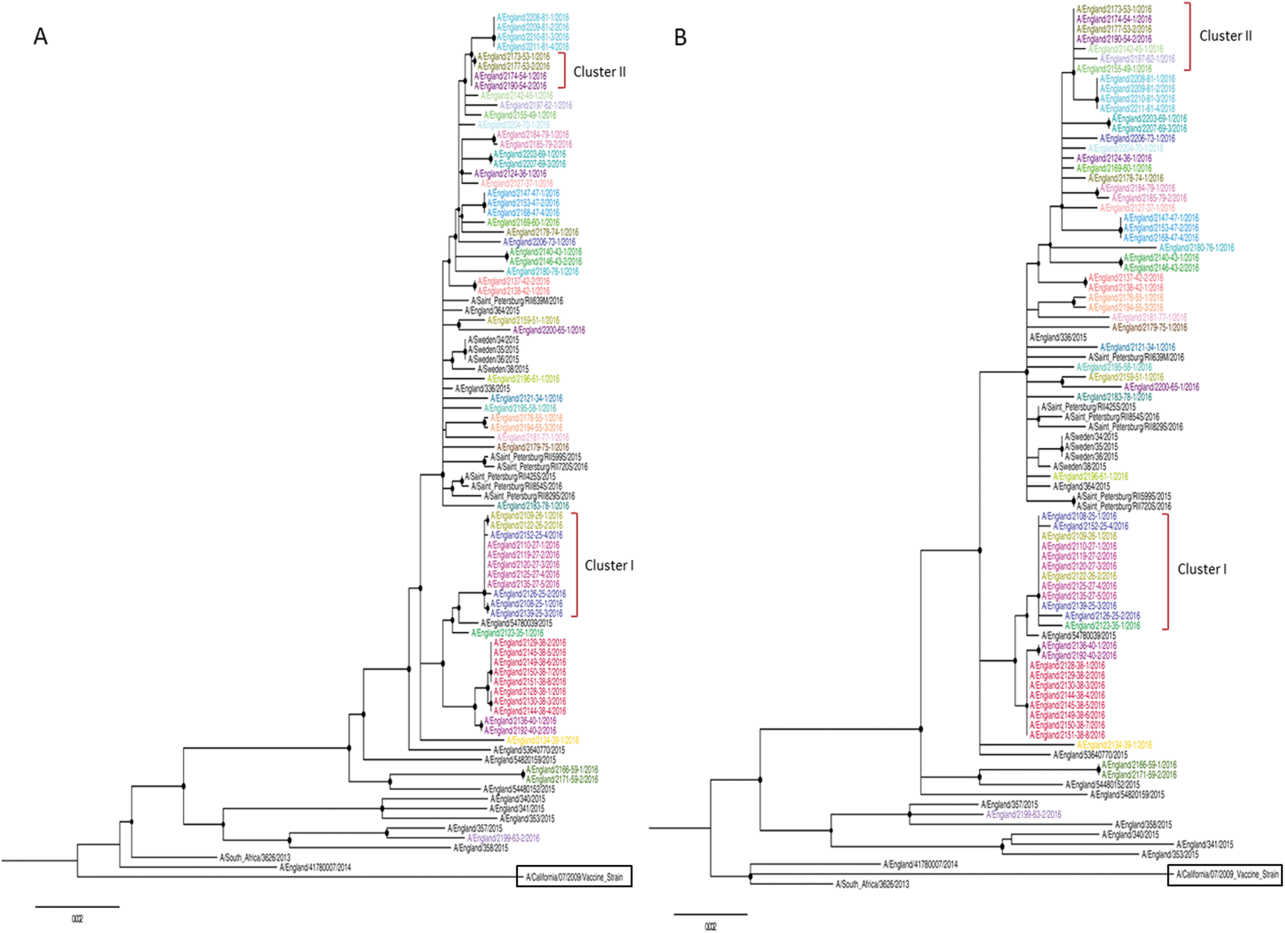
Maximum likelihood trees for (*A*) whole-genome and (*B*) HA/NA concatenated genes. The strains sequenced in this study are color-coded by individual patients. Bootstrap support >70% at nodes is highlighted using a black circle. The vaccine strain used in the formulation is highlighted in the box. Abbreviations: HA, haemagglutinin; NA, neuraminidase.

The amounts of sequence data and read depths for 80 samples, obtained from 38 patients, are shown in Table 2. Genome coverage and read depth correlated well with the inverse of the original diagnostic real-time polymerase chain reaction cycle threshold values (rtPCR) (Supplementary Figure 1). We successfully sequenced 65 samples, with a mean read depth >100X. The cut-off for generating whole genomes was cycle threshold 37 (approximately equivalent to 1000 gc/ml [genome copies/millilitre] of the original aspirate; Supplementary Figure 1). All samples were of the H1N1 genetic subtype 6B.1 and were phylogenetically distinct from the H1N1 vaccine strain used in the seasonal vaccine formulation (Figure 1A). An analysis of the 65 samples, from 36 patients, revealed near identical sequences (3 or fewer differences) at the consensus level between samples taken from the same patient. Phylogenetic analyses of these 65 genome sequences and of 24 other influenza A sequences circulating during the same season identified 2 monophyletic outbreak clusters, with high bootstrap values, occurring in the hospital (Figure 1A). Based on WGS, Cluster 1 was comprised of Patients 25, 26, and 27, and Cluster 2 contained Patients 53 and 54. Patients in Cluster 1 were all on the same ward at the time they became symptomatic, and this cluster had been previously identified by standard IPC procedures (Table 1). Cluster 2 was comprised of 2 patients, and this cluster had not previously been identified by IPC, as Patient 53 was initially noted to have been admitted with an acute infection from home. In addition, there was no phylogenetic evidence found for direct transmission between Patients 27 and 35 or between the 11 patients on the haematology/oncology wards. A phylogenetic analysis of HA/NA sequences alone generated a poorly supported tree, which identified both Clusters 1 and 2, but with low bootstrap values. Moreover, the tree failed to separate the sequences within these clusters from other closely related strains detected in the hospital (Figure 1B). The HA/NA tree of Cluster 1 included Patient 35, who had been readmitted 48 hours after discharge by the same medical team caring for Patients 25 and 26, albeit on a different ward, but who was not directly linked. By WGS, the virus isolated from Patient 35 was separated from Cluster 1 by an unrelated sequence. By HA/NA phylogenetic analysis, Cluster 2 included not only Patients 53 and 54, but also Patients 45, 49, and 62. In contrast, WGS identified only Patients 53 and 54 as part of a monophyletic cluster.

**Table 2.** Number of Clinical Samples Sequenced Successfully at Varying Genome Coverage and Depth of Coverage

	Number of Genomes Obtained
50% genome coverage at 1X mean depth	77
90% genome coverage at 1X mean depth	73
90% genome coverage at 100X mean depth	65

**Figure 2.**
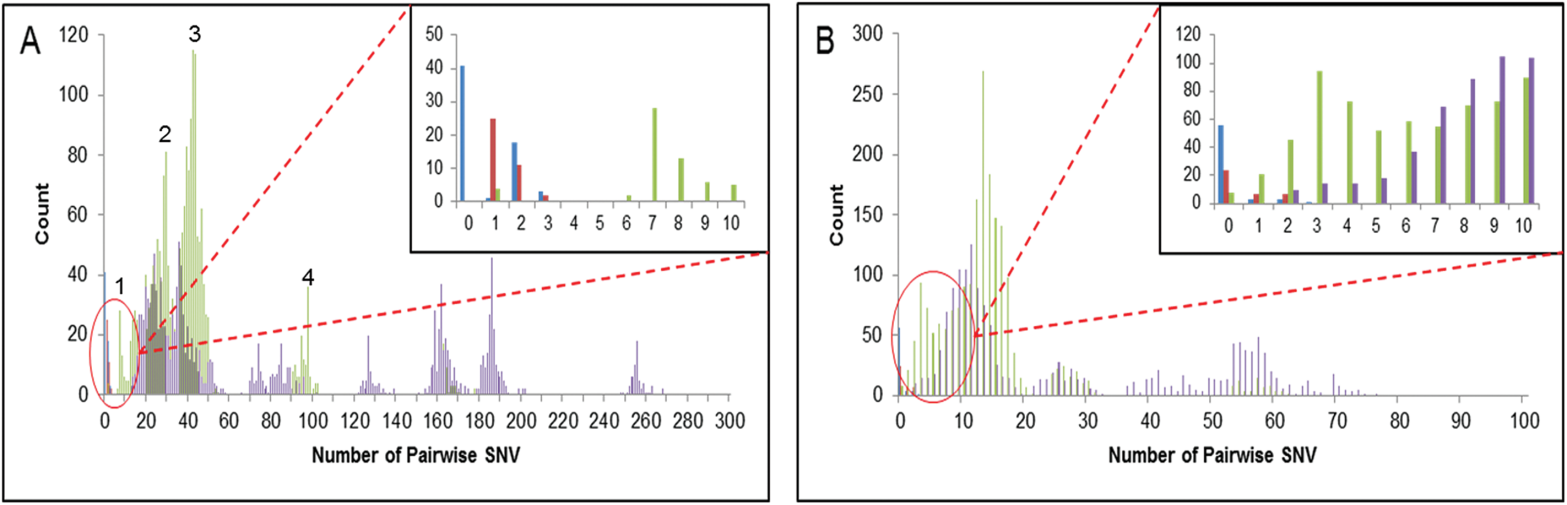
Pairwise SNV between strains in the study. The blue bars highlight variations within an individual patient; the red bars highlight variations between confirmed outbreak sequences; the green bars highlight variations between epidemiologically unrelated sequences within the hospital; and the purple bars highlight variations between strains circulating in Europe in the same season. Whole-genome SNV (*A*) and (*B*) HA/NA concatenated SNV. Abbreviations: HA, haemagglutinin; NA, neuraminidase; SNV, single nucleotide variations.

**Figure 3.**
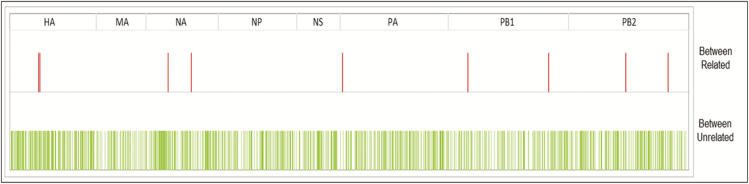
Variable sites highlighted across the genome between related (red) and unrelated (green) influenza A strains. The different segments and their respective boundaries are shown on top. Abbreviations: HA, haemagglutinin; MA, matrix protein; NA, neuraminidase; NP, nucleoprotein; NS, nonstructural protein; PA, PB1, PB2, polymerase protein.

To quantify further the differences between putative clusters of viruses and other viruses circulating during the same seasonal influenza epidemic, we calculated the pairwise genetic differences at the whole-genome level between strains circulating within the hospital and throughout Europe during the same season. For completeness, we compared the pairwise genetic differences for whole genomes with those obtained for concatenated HA/NA gene segments. The concatenated HA/NA genes, although among the most variable parts of the genome, revealed little differences in pairwise distances between epidemiologically unrelated strains co-circulating in the hospital during the same season: as shown in Figure 2B, blue (within-patient variation, calculated using longitudinal samples), red (strains from an IPC-confirmed outbreak cluster), green (epidemiologically unlinked GOSH strains), and purple (influenza database) pairwise distances overlap with each other. In contrast, at the whole-genome level, within-patient pairwise distances, calculated from samples collected longitudinally, were less than 3 across the entire genome, over periods ranging from 3 to 34 days (shown in blue in Figure 2A). The same pairwise variation was observed for viruses clustering as part of the confirmed outbreak in Cluster 1 (shown in red in Figure 2A). The 2 strains in Cluster 2 differed by 1 substitution (shown in green in Figure 2A). In Figure 3, we show the genome locations of variable sites, occurring within sequences taken from an individual or between sequences forming a known transmission cluster (red) and variable sites that separate epidemiologically unrelated genomes (green). The wide distribution across the genome and the intermingling of these 2 groups suggests that it is not possible to sequence any sub-genomic regions to distinguish within and between host variations.

**Figure 4.**
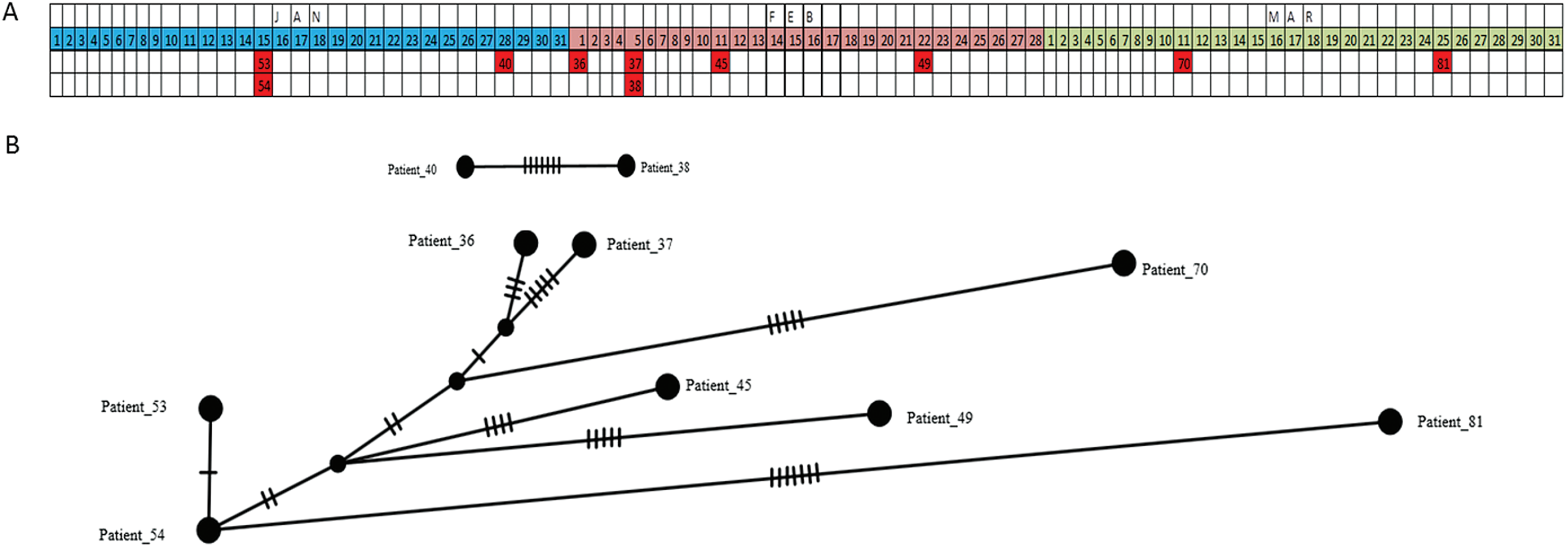
*A*, Table with dates of sampling for patients indirectly linked to cluster 2. *B*, Popart analysis of putative transmission chain of patients using a median joining network. Nodes with no labels are inferred nodes. The notches on each link between 2 nodes represent the number of changes between the 2 nodes at the whole-genome level.

The remaining GOSH strains, for which no direct epidemiological link could be found, fell into 4 normally distributed groups, labelled 1–4 (green in Figure 2A). In 3 of these groups (Table 1), 12 or more pairwise differences were intermingled, with unrelated sequences obtained from the influenza database (purple in Figure 2A). In contrast, members of the group with 6–11 pairwise changes were, with 1 exception, long-standing GOSH patients (Table 1). This group contained Patients 45 and 49, whose viral sequences, although distinct by WGS, clustered monophyletically with outbreak Cluster 2 (Patients 53 and 54) by HA/NA sequencing and also with Patients 40, 36, 37, 38, 45, 49, 70, and 81 (Table 1 and Figure 4). Figure 4A shows a timeline of when each patient within this group was sampled for the first time and the relationships between patients’ influenza sequences. From this, it seems that Patients 38 and 40 were potentially linked to each other, but not to the others. Both had visited GOSH as outpatients within a week of each other, but otherwise had no identified link (Table 1). Patients 36, 37, 45, 49, 70, and 81 all appeared to have potential links to outbreak Cluster 2 (Patients 53 and 54). On further analysis (Table 1 and Figure 4B), we found evidence for 3 putative transmission chains. Patients 53 and 54 presented on the same day on different wards, but had no direct contact. It is, therefore, likely that they were infected from a common source. Since Patients 53, 36, and 37 had all attended the outpatient department within 3 days of each other, all 3 could have been infected by this source. While Patient 53 became infected within 48 hours of attending as an outpatient, Patients 36 and 37 did not present until several days (nearly 2 weeks) later, suggesting that infection may first have occurred in a family member. Patients 54, 49, and 81 all acquired influenza whilst inpatients on the surgical wards. It is possible, therefore, that the staff member who infected Patient 53 also worked on the surgical wards. Thereafter, spread from this staff member or from Patient 54 to staff or families on the surgical wards may have resulted indirectly in transmission to Patients 49 and 81. Of interest, Patients 53, 54, and 49 were all private patients, who were particularly likely to have been cared for by the same staff. No link could be found between Patients 45 or 70 and other patients in the putative transmission chain. Patient 45 was an externally referred sample from a patient known to GOSH. Although there was no record that this patient had visited the hospital recently, it is possible that he/she had an unrecorded ward or outpatient visit. Patient 70 was not known to GOSH and presented with community-acquired influenza.

Patient 62, whose HA/NA sequence, but not the WGS, clustered with outbreak 2, had pairwise distances of ≥12 (Table 1). This child had briefly been an inpatient on the same ward as Patient 54 when the latter first became unwell, but had not had contact with GOSH since then and did not present until 6 weeks later. Similarly, while the HA/NA sequence from Patient 35 clustered with Patients 25, 26, and 27 (outbreak 1) and he/she was cared for by the same team, he/she had pairwise distances to other GOSH sequences of ≥13.

## DISCUSSION

Our data suggest that HA and NA sequencing alone do not adequately discriminate directly transmitted strains from those that are co-circulating in the same location. A previous study, which concluded that HA and NA sequencing was adequate, differed from ours in that it used sequencing to confirm well-founded links, only sequencing viruses from subjects suspected to be part of 2 outbreaks [14]. While this approach effectively distinguished the 2 outbreaks from each other, our study differed, in that we aimed to uncover previously unsuspected transmissions. Our approach revealed strains with identical HA and NA genes circulating in the same hospital population, as part of the same epidemic. In contrast, a phylogenetic analysis of WGS supported the direct transmission between Patients 25, 26, and 27, but excluded direct transmission to Patient 35, who had, by standard IPC methods, been tentatively linked to the outbreak. The closeness may, however, represent indirect transmission via other patients, relatives, or staff.

Applying standard influenza mutation rates of 0.09 mutations/genome/day [22] to the samples collected longitudinally from the same individuals suggests that 3.18 substitutions would have been expected over the period of 34 days during which samples were collected. This figure fits well with the 0–3 substitutions identified in this group. WGS demonstrated that the epidemiologically supported transmissions in Cluster 1 differed by single nucleotide polymorphism (SNP) numbers similar to those seen from within-patient, longitudinally collected samples (ie, 1–3 SNPs). Phylogenetic and pairwise analyses also supported the second cluster, as directly linked cases (Figure 2A). A retrospective analysis confirmed Patient 54, although presenting on the same day as Patient 53, was influenza positive on admission and was admitted to a different ward. Patients 53 and 54 had, therefore, not been considered linked by IPC methods. However, further investigation showed that Patient 54 had attended an outpatient unit run by the same medical team 2 days prior to their acute infection, raising the possibility that Patients 53 and 54 were infected by a common source. The need for WGS is further supported by our failure to identify clusters of variable sites that would enable more limited sequencing of subgenomic fragments to replace WGS (Figure 3).

The phylogenetic data for the haemato-oncology patients among whom IPC investigations had suspected cryptogenic nosocomial transmission, indicated no evidence of transmission, and this was supported by the pairwise genetic distances between them. This confirms that no breakdown in IPC had occurred in this unit. We observed a population of viruses circulating among GOSH patients (Figure 2A), which had pairwise genetic distances that were closer than those seen for any database viruses (6–11 versus > 13 SNPs; Figure 2A). Although no direct links between the patients could be found, an examination of the case histories showed that inpatient or outpatient care by the same medical team during the preceding weeks linked 8/10 of these patients. The possibility that Patient 45, for whom no link to other patients could be found, but who was a long-term GOSH patient, had had an undocumented outpatient visit remains unproven. Our data suggests transmission by unsampled, intermediate sources that led to Patients 53 and 54 becoming infected. The pattern of transmissions, occurring over a period of time, suggests that the original source may also have infected other staff or family members and led, eventually, to the infection of Patients 36 and 37. There were 3 patients, Patients 54, 49, and 81, who acquired their infections in the hospital, resulting in prolonged stays; Patient 36, who may have acquired the infection indirectly through outpatient attendance, required extra corporeal membrane oxygenation. Thus, the failure to interrupt this cryptic transmission proved costly, both medically and financially.

The use of pathogen genomic data to confirm and uncover putative transmission links between patients has the potential to be a powerful tool to aid traditional IPC approaches. We have shown that that the sensitivity and specificity of information provided by whole influenza genomes for the identification of true nosocomial clusters is greater than from sequencing HA and NA alone. Equally as important, we have shown that phylogenetic and pairwise distance analyses of WGS were able to uncover probable cryptogenic nosocomial influenza transmission in 1 unit, while excluding a suspected breakdown of IPC in the haemato-oncology unit. As turnaround times and per sample costs further decline, next-generation sequencing has the potential for routine use in clinical settings [18]. As hybridization times reduce (now 1 hour), it will be possible to generate sequencing material faster. In addition, newer, third-generation sequencing technologies, such as the MinION from Oxford Nanopore [23], have the potential for even faster turnaround times and are currently being tested for efficacy in real-time genotyping in a hospital setting. Our own experience of high-throughput, targeted enrichment methods has proven WGS of influenza to be sensitive, generating whole genomes from 1000 copies/ml, and fast, with turnaround times of 3–4 days and a cost of about £100/sample. While the infrastructure costs for high-throughput WGS include the purchase of robotics, low numbers of samples can be processed by hand. Here, we show the effectiveness of WGS and pairwise genetic analyses to identify direct transmissions that are not detected by standard IPC methods. While intensive sampling of staff and relatives would also be valuable, the data from whole genomes may, ultimately, render this unnecessary for uncovering linked infections. Notwithstanding, more data from staff and relatives could be useful for supporting campaigns to improve the uptake of the influenza vaccine among immunocompromised patients, their families, and the staff caring for them. As genomic data becomes linked to records of patient and staff movement, identifying accurately and in real time where breakdowns in IPC are occurring can become reality, thus allowing focused intervention to where it will have most impact. Such measures are likely to improve patient health and prove cost saving.

## Supplementary Data

Supplementary materials are available at *Clinical Infectious Diseases online*. Consisting of data provided by the authors to benefit the reader, the posted materials are not copyedited and are the sole responsibility of the authors, so questions or comments should be addressed to the corresponding author.

ciz020_suppl_Supplementary-Figure-1

## Notes

***Acknowledgments.***The authors thank the Medical Research Council University College of London/University College of London Hospital Biomedical Research Centre–funded Pathogen Genomics Unit for generating the sequences.

***Financial support.***This work was supported by European Community’s Seventh Framework Program Pathseek.

***Potential conflicts of interest.***J. B. receives funding from the University College London/University National Institute for Health Research Biomedical Research Centre. All other authors report no potential conflicts. All authors have submitted the ICMJE Form for Disclosure of Potential Conflicts of Interest. Conflicts that the editors consider relevant to the content of the manuscript have been disclosed.
