# Supplementary material for: Whole-genome Sequencing Provides Data for Stratifying Infection Prevention and Control Management of Nosocomial Influenza A
Source: Clin Infect Dis. 2019 Apr 17;69(10):1649–56. doi: 10.1093/cid/ciz020 (PMC6821348; doi:10.1093/cid/ciz020)
Supplement: ciz020_suppl_Supplementary-Figure-1 [file ciz020_suppl_supplementary-figure-1.docx]

**Supplementary Fig 1.**

**Supplementary Fig 1:** Mean read depth and percentage genome coverage as a function of pathogen load (Ct).
